# Sonoelastography for Testicular Tumor Identification: A Systematic Review and Meta-Analysis of Diagnostic Test Accuracy

**DOI:** 10.3390/cancers15153770

**Published:** 2023-07-25

**Authors:** Derek Ka-Hei Lai, Ethan Shiu-Wang Cheng, Ye-Jiao Mao, Yi Zheng, Ke-Yu Yao, Ming Ni, Ying-Qi Zhang, Duo Wai-Chi Wong, James Chung-Wai Cheung

**Affiliations:** 1 Department of Biomedical Engineering, Faculty of Engineering, The Hong Kong Polytechnic University, Hong Kong, China; 2 Department of Electronic and Information Engineering, Faculty of Engineering, The Hong Kong Polytechnic University, Hong Kong, China; 3 Department of Materials, Imperial College, London SW7 2AZ, UK; 4 Department of Orthopaedics, Ruijin Hospital, School of Medicine, Shanghai Jiao Tong University, Shanghai 200240, China; 5 Laboratory of Prevention and Treatment of Bone and Joint Diseases, Shanghai Institute of Traumatology and Orthopaedics, Ruijin Hospital, School of Medicine, Shanghai Jiao Tong University, Shanghai 200240, China; 6 Department of Orthopaedics, Tongji Hospital, School of Medicine, Tongji University, Shanghai 200065, China; 7 Research Institute of Smart Ageing, The Hong Kong Polytechnic University, Hong Kong, China

**Keywords:** ultrasound elastography, malignancy, seminoma, scrotum, Leydig cell, Sertoli cell

## Abstract

**Simple Summary:**

Testicular cancer is a prevalent malignancy in young men aged 15 to 35 years. Sonoelastography is an emerging technique for distinguishing between non-neoplasms, benignities, and malignancies by characterizing the tissue stiffness of testes. This review provides a summary of studies on the diagnostic accuracy of sonoelastography for identifying benign and malignant lesions, as well as tumors and non-tumors.

**Abstract:**

The objective of this review was to summarize the applications of sonoelastography in testicular tumor identification and inquire about their test performances. Two authors independently searched English journal articles and full conference papers from CINAHL, Embase, IEEE Xplore^®^, PubMed, Scopus, and Web of Science from inception and organized them into a PIRO (patient, index test, reference test, outcome) framework. Eleven studies (*n* = 11) were eligible for data synthesis, nine of which (*n* = 9) utilized strain elastography and two (*n* = 2) employed shear-wave elastography. Meta-analyses were performed on the distinction between neoplasm (tumor) and non-neoplasm (non-tumor) from four study arms and between malignancy and benignity from seven study arms. The pooled sensitivity of classifying malignancy and benignity was 86.0% (95%CI, 79.7% to 90.6%). There was substantial heterogeneity in the classification of neoplasm and non-neoplasm and in the specificity of classifying malignancy and benignity, which could not be addressed by the subgroup analysis of sonoelastography techniques. Heterogeneity might be associated with the high risk of bias and applicability concern, including a wide spectrum of testicular pathologies and verification bias in the reference tests. Key technical obstacles in the index test were manual compression in strain elastography, qualitative observation of non-standardized color codes, and locating the Regions of Interest (ROI), in addition to decisions in feature extractions. Future research may focus on multiparametric sonoelastography using deep learning models and ensemble learning. A decision model on the benefits–risks of surgical exploration (reference test) could also be developed to direct the test-and-treat strategy for testicular tumors.

## 1. Introduction

Testicular cancer is one of the most common malignancies among young males between the age of 15 and 35, accounting for 60% of all cancer cases in these age groups [1]. In 2020, there were 74,500 new cases worldwide [2]. Northern European countries, especially Norway and Denmark, had the highest age-standardized incidence rates, which were 11.5 and 10.2 per 100,000 person-year, respectively [3]. Perilously, the incidence has been steadily increasing since the mid-20th century and has doubled in the past 30 years [4,5]. Based on the rising trend, researchers predicted that the incidence will continue to increase over the next few decades despite the fact that they were unable to identify underlying reason of growth [4,6].

Although testicular tumors are infrequent compared to other types of malignancies, they nonetheless impose a burden on patients and society. It has been the leading cause of cancer-related mortality and morbidity in these age groups among males [7]. In China (Beijing), patients spent US$1577.7 and US$100.7 on 18 inpatient and 143 outpatient visits, respectively [8]. In Germany, the follow-up expenses after Stage I seminomatous germ cell tumors per patient totaled EUR 4430 in 2015 [9]. Although the survival rate was high [10], survivors experienced long-term mental health issues, such as depression, anxiety, and distress [11,12,13,14]. In particular, males experienced emotional challenges because of the cultural emphasis on masculine identity, regardless of whether they were, consequently, fertile or infertile [12,15].

Testicular cancer could be one of several types of neoplasms based on the cell origin and age of presentation [7]. Germ cell neoplasia in situ has been recognized as the most common precursor to malignancy, which could be further classified into seminomas and nonseminomas [7,16]. Seminomas have a higher incidence rate than nonseminomas (55% to 60% vs. 40% to 45%) and a comparable incidence trend [17,18]. Seminomas showed a greater incidence rate between the ages of 35 and 39, but nonseminomas were more prevalent between the ages of 25 and 29 [17,18]. Despite an unclear pathogenesis, early diagnosis of testicular cancer could improve cure rates and reduce the chance of metastasis [19]. Delayed diagnosis (>10 weeks) could result in a reduction in survival rate [20] and double the treatment cost, comparing that at advanced and early stages [21]. In addition, both a diagnosis (identifying tumor from non-tumor) and differential diagnosis (classifying benign and malignant) of testicular cancer are equally important to facilitate the selection of appropriate treatment.

A physical examination is the primary method for evaluating potential testicular tumors [22]. Other diagnostic tests include ultrasonography, computed tomography (CT), positron emission tomography (PET), and tumor marker tests while histology is frequently used to confirm a diagnosis [22]. In fact, ultrasonography is one of the non-invasive instruments for a testis diagnosis [23]. Not only is it capable of assessing traumatic, vascular, neoplastic, and inflammatory problems, but it could also enable the detection of small, non-palpable lesions in clinical practice [24]. Conventional ultrasound modalities included B-mode and color doppler. B-mode ultrasound could identify the morphological information (size, shape, location) and the echogenic pattern of the lesion, whereas vascularity, an indication of malignancy, could be evaluated by color doppler ultrasound [25]. Nevertheless, they were believed to have low specificity [26] but can be improved by incorporating contrast-enhanced ultrasound (CEUS), which facilitates a better visualization of microvascularization through contrast agents [27,28].

The sonoelastography (or ultrasound elastography) is a relatively new ultrasound technique that measures and maps the physical properties of tissues under deformation (i.e., stiffness) [29]. A sonoelastography was also used to assess the liver [30,31], brain [32], and lymph nodes [33]; musculoskeletal diseases [34]; and athletic performance [35] in addition to the detection of various cancers or tumors [36,37,38]. The basic premise for using a sonoelastography to identify or classify testicular tumors is that malignant tumors are generally stiffer than benign lesions and normal testicular parenchyma [27]. Nevertheless, in practice, some incidental benign testicular lesions (e.g., Leydig cell tumors) could have similar tissue stiffness as surrounding tissue because of their vessel density [39]. Some malignant lesions could exhibit avascular patterns due to necrosis and fibrosis that influence the diagnosis [40]. Due to these challenges, we considered that the diagnostic accuracy of sonoelastography for testicular evaluation for tumors in clinical practice could be questionable.

To this end, the objective of this study was to summarize the methodologies and diagnostic performance of sonoelastography in identifying testicular tumors. The following review questions were to be addressed:

1. How well can sonoelastography distinguish a testicular neoplasm from a non-neoplasm (i.e., identify a tumor)?
2. How well can sonoelastography classify benign and malignant tumors?
3. What are the sonoelastographic determinants for the identification/classification? And how could they be obtained?

## 2. Materials and Methods

### 2.1. Eligibility Criteria

The search strategy referred to the PIRO layout (i.e., population, index test, reference test, and outcomes). We focused on diagnosing/screening testicular tumors using ultrasound elastography. The study design of the eligible articles was related to the diagnostic accuracy of these tests. There was no constraint on the type of testicular tumors or the choice of reference test. In addition, we did not control the eligibility based on the nature of the diagnostic classification. In other words, the diagnostic classification could be targeted at the identification of tumors (from non-tumors) or the differentiation between benign and malignant tumors. It could be on a per-patient, per-testis, or per-lesion basis.

### 2.2. Information Source

The first and second author (D.K.-H.L. and E.S.-W.C.) independently searched the literature in April 2023 from CINAHL (default field), Embase (title/abstract/keywords), IEEE Xplore^®^ (all metadata), PubMed (title/abstract), Scopus (title/abstract/keywords), and Clarivate Web of Science (topic field). Only English journal articles or full conference papers were selected. We did not limit the year of article publication.

### 2.3. Search Strategy

The search terms included those related to testicular cancer, ultrasound elastography, and outcome measures for diagnostics. Search terms on testicular cancer shall include those describing both testis (“testicul*”, “testes”, “testis”, “scrotum”, “scrotal”, “Leydig”, “Sertoli”, “Sertoli-Leydig”) and cancer (“carcinoma”, “malignan*”, “cancer*”, “lesion*”, “tumor*”, “tumour”, “mass*”, “seminoma”, “nonseminoma”). In addition, the search algorithm shall include either “sonoelastograph*” or “SWE” or terms appearing in both ultrasound (“ultrasound”, “sonograph*”, “ultrasonograph*”, “echograph*”) and elastography (“elastograph*”, “tissue stiffness*”, “modulus measure*”) domains.

Outcome measure related keywords included “accuracy”, “sensitivity”, “specificity”, “precision”, “recall”, “PPV”, “NPV”, “positive predictive value”, “negative predictive value”, “F1-score”, “ROC”, “AUROC”, “receiver-operating”, “Youden’s index”, “Youden index”, “Matthews Correlation Coefficient”, “Balanced Classification Rate*”. The review protocol and search terms/algorithms could be found in the registered PROSPERO document (reference number: CRD42023422540).

### 2.4. Study Selection Process

Inclusion criteria included: (1) original research articles; (2) published in English; (3) published in either journal articles (including in-press articles), preprints, or conference full papers; (4) articles that applied ultrasound elastography, either alone or with other ultrasound modalities; (5) articles that identified or classified testicular tumors; (6) articles reported classification performance using accuracy-related outcome measures, e.g., accuracy, sensitivity, specificity, area under receiver-operative characteristics (ROC) curve etc.

Exclusion criteria included: (1) article types that were neither journal articles/preprints nor conference full papers, for example, review articles, perspective and commentary articles, conference abstracts, book chapters, and patents; (2) articles that did not involve the identification of malignancy, e.g., only targeting cysts, focal infarction, or microlithiasis; (3) classification performance evaluated on non-human data, e.g., animal model, phantom, or simulation data.

### 2.5. Data Collection and Extraction

The first author screened the title, abstracts, and full texts, which were checked by the second author. Any disagreement was resolved by consensus with the corresponding authors. The data synthesis was based on the PIRO framework. The study characteristics were tabulated and summarized into population (with sample size, mean age, sampling approach, patient source, and referral indications), index test (instruments, comparison, and diagnosis methods), reference test for true positives and true negatives, and outcomes of classification performance.

### 2.6. Methodological Quality Assessment

Risk of bias and applicability of the included studies were assessed using the Quality Assessment of Diagnostic Accuracy Studies—2 (QUADS-2) [41] by the first author (D.K.-H.L.) and verified by the corresponding authors (D.W.-C.W. and J.C.-W.C.). The instrument covered four domains with seven question items, including the risks of bias and applicability concerns of patient selection, index test, reference standard, in addition to applicability concerns of flow and timing. Each item would be rated as “yes”, “no”, and “unclear”. The risk of bias and applicability concerns graph and summary were visualized using Review Manger (RevMan) 5.4.1 (The Cochrane Collaboration, 2020).

### 2.7. Meta-Analysis

Meta-analyses of test performance were done separately for the classification of tumor/non-tumor (i.e., neoplasm/non-neoplasm) and benign/malignancy lesions using ultrasound elastography alone (i.e., excluded those findings that integrated with B-mode or Doppler ultrasound). For studies that applied multiple variables, we decided to investigate the visual elastographic score since the scale and threshold values were predetermined and quite consistent across studies. In cases when reported results contradicted the reported data, the data information prevailed.

Random-effect bivariate models (multivariate meta-analyses) were utilized to calculate the summary estimates (or summary point), pooled sensitivity, and specificity, which were illustrated using coupled forest plots. Hierarchical Summary ROC (HSROC) plots were also used to create a summary line to complement the estimations of summary points where appropriate [42]. Since *I^2^* statistics or Cochran’s *Q* tests were not suitable for meta-analysis of test accuracy, the heterogeneity among studies was evaluated by visual impression of the variability of sensitivity and specificity in the coupled forest plot and HSROC plot [43]. Methodological characteristics were added as covariates for subgroup analysis (SE: qualitative analysis through color codes, SE: semi-quantitative analysis using scoring systems, and SWE). Publication bias, in terms of small-study effects, was evaluated using the Deek’s test for funnel plot asymmetry, a weighed linear regression on the log diagnostic odds ratio on the inverse of the squared effective sample size using the effective sample size as weights [44].

The coupled forest plots were plotted using the Review Manager (RevMan) 5.4.1 (The Cochrane Collaboration, 2020). For better visualization, the HSROC plot was generated using an interactive web-based tool [45,46] that was built by the R statistical package and libraries (R project for Statistical Computing, Vienna, Austria).

## 3. Results

### 3.1. Search and Study Selection Results

**Figure 1:**
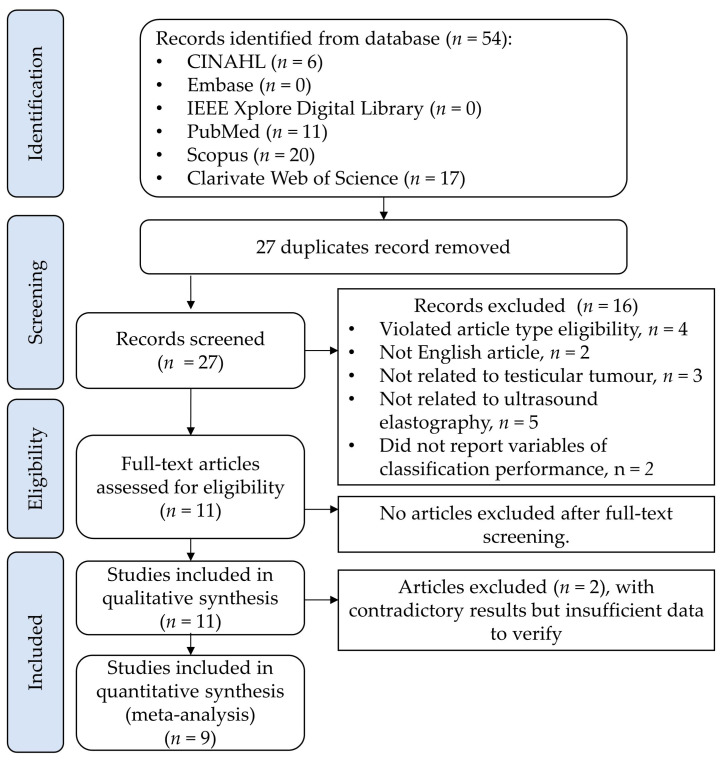
PRISMA flowchart for the systematic review detailing database searches, studies screened, excluded, and retrieved.

As shown in Figure 1, the raw search identified 54 hits, and 27 records were eligible for screening after removing the duplicates. A primary screening based on the article title, abstract, and keywords excluded 16 studies with reasons (violated the inclusion criteria of the article type, *n* = 4; not an English article, *n* = 2; not related to testicular tumor, *n* = 3; not related to ultrasound elastography, *n* = 5; did not report outcome variables of classification performance, *n* = 2). No articles were excluded after full-text screening. Finally, eleven papers (*n* = 11) were eligible for data synthesis [47,48,49,50,51,52,53,54,55,56,57]. Three studies presented contradictory results and two were unverifiable [55,57]. Therefore, nine studies (*n* = 9) furthered the meta-analysis.

### 3.2. Qualitative Synthesis

#### 3.2.1. Population

Among the 11 studies, nine of them gave clear information on both the number of patients and the number of lesions. As shown in Table 1, assuming all unspecified cases were unilateral lesions, the review involved data from a total of 1027 patients with 1306 testes. It shall be noted that Goddi et al. [50] accounted for multiple lesions per testis while the other studies were assumed to examine a single lesion per testis. The mean or median age of the studies ranged from 30.0 to 43.2, excluding Goddi et al. [50], which did not provide the age information of eligible patients.

Most of the studies (10/11) adopted a consecutive sampling approach. Four studies were retrospective while five studies were prospective. Additionally, only five studies (*n* = 5) explicitly stated their patients were sourced from clinical sectors. The referral indications of the attended patient included testicular or scrotal pain and abnormality, infertility, andrological screening, suspicion of testicular tumor, and follow-ups on indeterminate ultrasound findings. Depending on the scope of the classification, the inclusion and exclusion criteria among studies were different. Benign tumors were identified from the studies, including lipoma, adrenal rest tumor, papillary cystadenoma, and Sertoli cell tumor. Leydig cell tumors were the most frequent benign neoplasm [49,51,52,54] despite the fact that some studies attempted to identify malignant Leydig cell tumors [47,52]. Screening malignant tumors was one of the major goals, and these tumors included germ cell tumors (seminomas, non-seminomas, and mixed non-seminomas), teratomas, embryonal carcinoma, lymphomas, burned-out testicular tumors, sex cord stromal tumors, and metastasis from adrenal tumors. On the other hand, some studies endeavored to distinguish between tumors (neoplasms) and non-tumors (non-neoplasms). Patients without tumors (neoplasms) might suffer from different forms of cysts (epidermoid and dermoid), inflammations (epididymo-orchitis, orchitis), scarring/fibrosis, abscesses, microlithiasis, and hematomas/granulomas. Table 2 summarizes the testicular/scrotal problems that are classified into non-neoplastic (non-tumor), benign, and malignant.

#### 3.2.2. Index Test

While elastography and B-mode ultrasound were frequently combined [52,53,55,57], several studies reported separate instruments and involved other kinds of ultrasound instruments [47,48,49,50,51,54,56], as shown in Table 3. In addition to B-mode and Doppler ultrasound that routinely looked for echogenicity and vascularization from a possible malignancy, two studies used contrast-enhanced ultrasonography (CEUS) [48,49], which combined ultrasound imaging with patients injecting a contrast agent to improve the visibility of blood vessels and tissues. Auer et al. [48] administered 2.4 mL of microbubble-based contrast agent (SonoVue, Bracco, Vienna, Austria) intravenously followed by a saline flush while that of Corcioni et al. [49] was 4.8 mL of second-generation contrast media (SonoVue, Bracco, Milano, Italy). In addition, Corcioni et al. [49] followed the CEUS procedures based on the European Federation of Societies for Ultrasound in Medicine and Biology Guidelines and the Recommendations for the Clinical Practice of CEUS in Non-hepatic Applications [58].

The morphological information of the lesions, including their size (diameter, volume) [47,49,50,52,53,56,57], shape [50], location [49], number [54], lesion margin (edge) [49,54], and presence of microlithiasis and calcification [51,52,54], was evaluated in previous studies using B-mode ultrasound. Goddi et al. [50] divided the lesion shape into nodular and pseudo-nodular with sizes of <5 mm, 6–10 mm, and <11 mm, whereas Corcioni et al. [49] examined the lesions’ margins for sharpness or irregularity. The volume of the lesions was calculated by approximating them as ellipsoids [47]. In addition, the echogenicity data in B-mode indicated if the mass was more solid and dense, representing the presence of a possible malignancy. It was frequently classified as hypogenic, hyper- or isogenic, and heterogeneous [47,48,49,51,52,53,54,56,57], and Konstantatou et al. [51] also sought to identify cysts though echogenicity.

Strain elastography (SE) and shear-wave elastography (SWE) are two common modalities of sonoelastography. In this review, nine studies (*n* = 9) utilized SE, and two studies (*n* = 2) utilized SWE. For SE, external pressure was applied by a transducer manually to exploit tissue deformation, which was used to estimate the tissue strain and thus elasticity [59]. During the process, the testes would be fixed to a scrotum plate to facilitate optimal positioning and alignment. The operators applied a gentle vertical pressure on the testis freehand and adjusted the pressure according to the real-time visual indicator of the instrument, which relied on experience [60]. Goddi et al. [50] supplemented that the visual indicator would report a pressure intensity of one to five “Hitachi units”, and they maintained the pressure at a level of three. To improve the reliability, some studies attempted multiple acquisitions [48,53] or adopted a multi-compression imaging technique to improve the signal-to-noise ratio [61,62]. On the other hand, SWE was a relatively new technique to quantify the elasticity by producing an acoustic pulse and measuring the speed at which the pulse’s shear wave propagates, which is dependent on the tissue stiffness [63]. The technique could minimize the variability of irregular manual compression [63]. In both cases, the tissue stiffness could be color-coded and mapped onto spatial images of the B-mode for better visualization [63].

The analyses of ultrasound elastography were described as qualitative and semi-quantitative [52,56]. Elastograms displayed a non-standardized, arbitrary gradient of color hue from red, green, and blue, signifying soft, intermediate, and hard tissues, respectively, in terms of the gauged strain. Therefore, by definition, it is not a quantitative indicator of stiffness/hardness (i.e., Young’s modulus) [64]. Nonetheless, some studies diagnosed malignancies or tumors based on the color codes or chromatic values and the radiologists’ decisions [47,48,49,53,57]. In contrast, a semi-quantitative technique was presented by visual scoring systems. Goddi et al. [50] and Pozza et al. [52] implemented the Itoh’s 6-point visual elastographic score system, which was originally used for diagnosing breast cancer [65]. If the lesion exhibited uniform strain for the entire lesion, it would receive a score of one in the system. A score of two and three suggested that strain appeared in most of the lesion and at the periphery except the center, respectively. A score of four showed no strain (i.e., hardest) in the whole lesion while a score of five extended the absence of strain region to the surrounding region [65]. A cut-off value of three corresponded to potential malignancy [65]. Schröder et al. [56] augmented the method by including a score level, “chaos”, characterized by a multi-colored elastogram pattern and manifesting possible neoplasticity, in addition to the differences of visual elastographic scores between the lesion and healthy tissue. There were also other scales, including Patel’s 3-point scoring system [66] that collapsed the Itoh’s scale and Yusuf’s 6-point visual elastography score system [67]. A score of one in Yusuf’s system was characterized by an all-green region with some red spots. A score of two showed a completely green region while a score of three might include some small blue spots. Images scored four exhibited a blue center but were green at the periphery. The lesion would be completely blue for those scoring six and might include little green and red spots in the center in the case of scoring five. The cut-off was also three for malignancy [67].

The strain ratio (also known as deformation quotient) was an additional semi-quantitative metric used to evaluate the likelihood of neoplasms by computing the average strain ratio between lesions and normal tissues. In order to do this, the Region of Interest (ROI) of the lesions and surrounding normal tissues would be outlined by overlaying the B-mode images. Goddi et al. [50] employed rectangular boxes for the ROI, but Rocher et al. [54] and Roy et al. [55] adopted spherical/circular boxes (also named as Q-box). ROI could also be manually delineated using free curves [51,52]. The threshold value for the strain ratio was determined using an ROC analysis [51,56]. Some variations on the methodology were also proposed, including the use of a maximum value [54], average value [54], and the degree of filling of the ROI [55]. Furthermore, Schröder et al. [56] proposed the use of Q-size, which was the ratio of the elastogram and B-mode ultrasound measured lesion size. Under the premise that neoplastic lesions appeared to be larger on the elastogram due to peritumoral fibrosis, a Q-size of >1.05 was deemed abnormal [56].

The index test depended on subjective judgements and empirical experience of the radiologists, which may be prone to bias. Table 3 reports the number of radiologists or uroradiologists who did the index tests (elastography) and their years of experience. If the operator and the reader were not the same person, the reader’s information was shown. Six studies (*n* = 6) utilized a single operator/reader [47,49,53,54,56,57], and the remaining five studies (*n* = 5) employed a minimum of two and a maximum of three operators/readers [48,50,51,52,55]. The year or experience varied from 5 to more than 20 years. Auer et al. [48] and Roy et al. [55] included an additional radiologist for data validation and performed an inter-rater analysis. In addition, four studies (*n* = 4) clearly mentioned that the index test was blinded from the reference test [47,48,51,52]. Nevertheless, three studies (*n* = 3) indicated that there were distinct operators and readers and that the readers might have been blinded to patient data and reference test although this was not clearly stated [51,52,55].

#### 3.2.3. Reference Test

Surgical exploration with a histopathological analysis was the most common and standard approach (reference test) to confirm tumors or malignancies (Table 4). Different guidelines were proposed to perform the histopathological analysis [68,69,70]. Nevertheless, due to the risks of surgery, the majority of studies (8/11) conducted the histopathological investigation only if tumors/malignancies were suspected in the ultrasound evaluation [47,48,50,51,52,53,54,57] and some patients declined surgery, thus a histopathological analysis [49,52]. Only two studies verified both positives and negatives with a histopathological analysis [49,56] while Corcioni et al. [49] excluded patient data if histopathological investigation was not done.

In most of the cases, the negative test findings were confirmed by clinical follow-ups. While Pozza et al. [52] and Reginelli et al. [53] did not define the duration of follow-up, the minimum follow-up period may be one month [57], three months [47,48,50], six months [50], or until resolution or non-progression was confirmed [51]. If inflammation or infarction was anticipated, follow-ups would be more frequent in the first few sections [47,48]. During follow-ups, a negative test result was validated if the lesion was stabilized, reduced, vanished, or its vascularity decreased [47,48]. In addition, Pozza et al. [52] followed and reported a more detailed protocol [39,71]. They consider the lesion to be non-neoplastic (e.g., Leydig cell hyperplasia, segmented ischemia, and fibrosis cysts) if there were any multiple and/or non-vascularized lesions, whereas any lesions that were single, entirely solid, hypoechoic, with internal vascularization that lasted for at least 18 months were termed benign neoplasms. In contrast, the result may be confirmed by the diagnosis of another condition. For example, recovery after antibiotic treatment [55,57], history of trauma with reduced lesion size during follow-ups [57], scar (fibrosis) from testicular biopsy [57], and biomarkers [52,53,54,55].

#### 3.2.4. Outcome Measures and Classification

Test performance was evaluated by comparing the index test against the reference test through a 2-by-2 contingency table using TP, TN, FP, FN. Accuracy is defined as the proportion of accurate index test results confirmed by the reference test out of the total number of tests. The ROC curve displays the continuum of all threshold values for classification on the function of positive rate versus the false positive rates, which is an indicator of discriminative capability. The other outcome measures, derived from the contingency table, are illustrated in the Equations (1)–(4). In contrast, clinicians favored the use of more clinically directed variables, such as diagnostic odds ratio (DOR) and likelihoods, as shown in Equations (5) and (6), because they may have difficulty understanding test performance-related variables [72].

Sensitivity (Sn) or Recall = TP/(TP + FN)(1)

Specificity (Sp) = TN/(TN + FP)(2)

Positive Predictive Value (PPV) or Precision (Pc) = TP/(TP + FP)(3)

Negative Predictive Value (NPV) = TN/(FN + TN)(4)

Positive Likelihood Ratio (LR+) = Sn/(1 − Sp)(5)

Negative Likelihood Ratio (LR-) = (1 − Sn)/Sp(6)

For classification, six studies (*n* = 6) aimed to identify tumors or neoplasms [47,51,52,55,56,57], assuming that classification of tumors and non-tumors was equivalent to that of neoplasms and non-neoplasms. Seven studies (*n* = 7) sought to classify malignant and benign tumors [48,49,50,51,52,53,54]. Leydig cell tumors and burnout tumors were also addressed by the research undertaken by Corcioni et al. [49] and Rocher et al. [54]. In addition, it should be noted that some studies included multiple comparisons.

For the identification of neoplasms/tumors, using strain elastography alone, Aigner et al. [47] appeared to have the best performance by analyzing the tissue stiffness through the color codes (Table 5). They reported an accuracy of 94% and a sensitivity of 100% [47]. The lowest sensitivity values were 69.3% and 58.7%, reported by Pozza et al. [52], using the strain ratio and visual elastographic score. Schröder et al. [56] assessed several strain elastography variables and achieved about 80% and 90% accuracy and sensitivity, respectively, except for Q-size. However, the specificity was less than half using strain elastography [56]. With SWE ultrasound, Roy et al. [55] detected tumors at a sensitivity of 82% by measuring the degree of ROI filling and established the threshold by ROC analysis. In addition, Rocher et al. [54] successfully increased the accuracy from 79.8% to 86.5% by considering the observed features of B-mode and Doppler with SWE ultrasound.

For the classification of benign and malignant tumors, the range of sensitivity using strain elastography with the visual elastographic score was between 80.7% and 87.5% [50,51,52] but that using the strain ratio seemed to be worse, ranging from 59.4% to 74.2% [51,52] (Table 5). Though observing the color codes of strain elastography, Auer et al. [48] and Reginelli et al. [53] attained accuracies of 78.2% and 87.0%, whereas Reginelli et al. [53] enhanced the accuracy to 90% by including the observed features of B-mode and Doppler ultrasound [53].

#### 3.2.5. Study Quality (Risk of Bias and Applicability)

**Figure 2:**
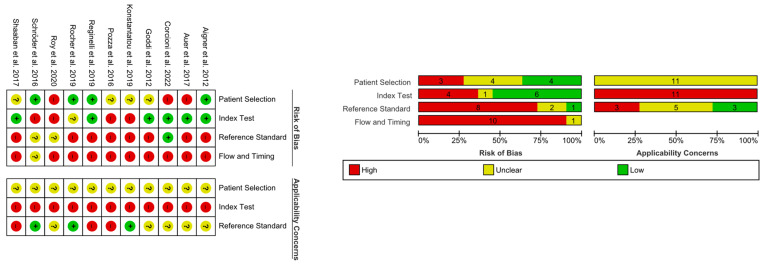
QUADAS-2 assessment on the risk of bias and applicability concerns of the included studies [47,48,49,50,51,52,53,54,55,56,57]. Left Figure: Red circle with negative sign denotes high risk. Yellow circle with question mark denotes unclear risk. Green circle with positive sign denotes low risk.

In terms of bias risk and applicability, the included studies were of rather poor quality. Given the seven questions, five studies received two points, four received one point, and two received no points (Figure 2). More studies (6/11) earned points for addressing the risk of bias of the index test, but lost points for the items, the risk of bias of flow and timing, and the applicability concerns of patient selection and index test. The former was contributed by the fact that the index tests were conducted before the reference tests in addition to the pre-determined thresholds. The risk of bias in flow and timing was attributed to the uncertain latency between the index and reference tests, inconsistent reference tests for positives and negatives, and the exclusion of patient data after enrollment (e.g., loss of follow-ups, poor data quality). Applicability concerns over the patient selection stemmed from the vast range of testicular diseases (Table 2), excluding patients who had an “obvious” diagnosis, whilst those of the index test were influenced by the subjectivity and variability of the index test, respectively.

### 3.3. Quantitative Synthesis

#### Meta-Analysis

**Figure 3:**
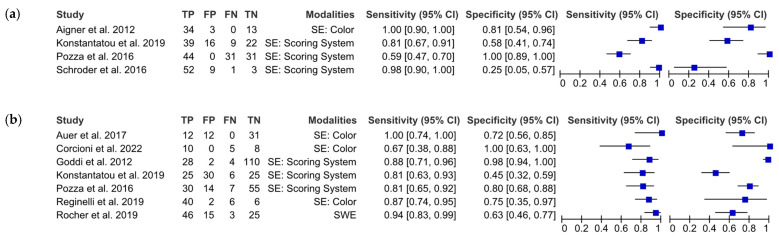
Coupled forest plot for studies classifying: (**a**) non-neoplasm and neoplasm and (**b**) benignity and malignancy [47,48,49,50,51,52,53,54,56]. SE: strain elastography; SWE: shear-wave elastography.

The pooled estimates with subgroup analysis are shown in Table 6 and the coupled forest plots for non-neoplasm vs. neoplasm (i.e., non-tumor vs. tumor) (*n* = 4) and benignity vs. malignancy (*n* = 7) are illustrated in Figure 3. Note that two studies of the former classification were excluded from the meta-analysis due to unverifiable contradictory results. In addition, based on the meta-analysis findings, we opted not to provide the result of Deek’s test for funnel plot asymmetry since it might be misleading in the presence of substantial heterogeneity and because the number of studies was less than 10 [73].

**Figure 4:**
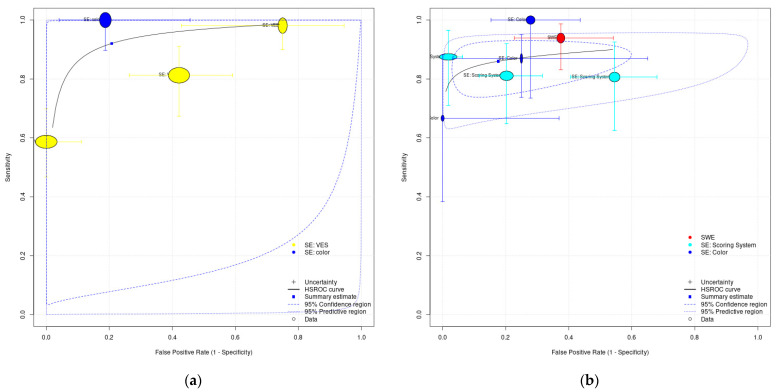
Hierarchical Summary Receiver-operating characteristics (HSROC) curve for studies classifying: (**a**) non-neoplasm and neoplasm and (**b**) benignity and malignancy. SE: strain elastography; SWE: shear-wave elastography.

For the meta-analysis for identifying neoplasms from non-neoplasms, the pooled sensitivity and pooled specificity were 92.1% (95%CI, 62.8% to 98.7%) and 79.2% (95%CI, 26.2% to 97.6%), respectively. Nevertheless, substantial heterogeneity was observed in the coupled forest plot. The HSROC plot especially showed that the study-level data points were dispersed in the ROC space with a large area of 95% prediction interval (Figure 4). Therefore, we decided not to conclude this finding.

For the meta-analysis for identifying malignancy from benignity, the boxes of the coupled forest plot were nearer with a pooled sensitivity of 86.0% (95%CI, 79.7% to 90.6%), and the study-level data points were relatively closer to the summary ROC curve. The slanted 95% confidence and prediction region in the HSROC plot demonstrated that substantial heterogeneity was observed for the specificity, with a pooled specificity of 82.4% (95%CI, 60.4% to 93.5%). Since the study-level data points with a subgroup did not seem to be clustered, we believed that the sonoelastography technique and approach could not or could not adequately explain the source of heterogeneity.

## 4. Discussion

The sonoelastography produced a promising test performance in detecting tumors or malignancies, as indicated by the high pooled sensitivity and specificity in our meta-analyses. In addition, our data synthesis revealed that multiparametric ultrasound (i.e., integrating different ultrasound modalities) might improve the test performance, as advocated by the other literature as well [63,74]. The sonoelastography did, however, suffer from a few methodological and technical issues that led to bias and application difficulties. In fact, it was considered a qualitative or semi-quantitative method. SE evaluations rely on the expertise of operators or radiologists in manually applying an adequate probe-compression and formulating a diagnosis based on the observations of the arbitrary color codes, either qualitatively or with the help of a semi-quantitative ordinal scale. Though less popular, SWE did not have the variation problems of manual compression and color code observation. Alternatively, the variances in manual compression could be minimized using acoustic radiation force impulse (ARFI) elastography [75], a force- and position-controlled probe [76], or robotic control [77].

The characterization of lesion features was another challenge. First, elastography systems, both SE and SWE in this review, were two-dimensional. To enhance the assessment of lesion morphology and volume, three-dimensional ultrasound elastography was essential and was proven feasible [34,78]. In addition, our data synthesis revealed that several studies sought to compare the performance of tests employing different feature variables. The traditional machine learning approaches could discriminate between benign and malignant masses by selecting, combining, and weighing a set of predetermined features [79]. It could be further improved by using deep learning models that enable automated segmentation of lesion regions (ROI) and learn the features automatically from raw input [80] despite the greater demand on the amount of data to achieve good performance [81]. Furthermore, ensemble learning can optimize the performance of multiparametric ultrasound or multimodal medical imaging [82,83].

Verification bias due to an imperfect reference standard was one of the key concerns. Not only were patients not receiving the same reference standard, but surgical exploration and histological examination (i.e., gold standard) were only performed to verify positive cases. Negative cases were followed by “active surveillance”, but there was no consensus on the minimal frequency and duration of follow-up. In addition, the procedures for verifying negatives were ambiguous. For instance, some studies did not describe the procedure and decision-making process of the follow-up. Some studies focusing on the detection of malignancy considered a “bad” follow-up to be a benign case and a “good” (resolved) follow-up to be non-neoplastic, with no condition driving a false negative (i.e., undetected malignancy by the index test). Moreover, the techniques for establishing differential diagnoses on a broad spectrum of benign and non-neoplastic diseases was also vague, subjective, and physician-dependent, which posed applicability concerns and the potential of misclassification. While we acknowledged that the reference standard (follow-up and differential diagnosis) in the current situation was as good as it is going to be to support a meta-analysis [84], a latent class model with Bayesian hierarchical modeling may be used to estimate the true disease status under certain assumptions [85,86]. Using the findings of meta-analyses, future research should consider establishing a decision model on the benefits-risks of surgical exploration and direct the test-and-treat strategy [87,88].

There were some limitations in this review. A language bias might be contributed by including solely English articles. Moreover, our search was limited to the stated databases and publications, such as journal papers and conference full papers, which may have resulted in evidence selection bias. Some studies investigated the differences in ultrasonic elastographic characteristics for differential diagnoses [66,89,90,91,92,93] but did not evaluate the diagnostic accuracy and were thus excluded from this review. On the other hand, in the meta-analyses, we did not analyze all variables for studies that employed multiple variables (e.g., using strain ratio). A sensitivity analysis was also not conducted on the methodological quality since most of the included studies did not perform well. Nonetheless, it shall be noted that doing meta-analyses on studies with a high risk of bias might result in estimates that are seriously deceptive and shall be interpreted with caution [94]. Our subgroup analysis of the modalities and techniques of sonoelastography could not or could not fully explain the heterogeneity. In addition to methodological characteristics, clinical heterogeneity, including lesion size, morphology, differential diagnosis, and other factors/features, may have a major impact on the outcomes of meta-analysis results, particularly for strain elastography that is operator-dependent. The same problem, as well as the small number of papers eligible for the meta-analysis (<10), precluded us from assessing the publication bias in terms of small-study effects [95], which frequently occurred in imaging diagnostic accuracy studies [96]. Lastly, our review centered on the test performance of ultrasound elastography (alone) to identify tumors or malignancies. Several studies attempted to compare or integrate multiple ultrasound modalities or techniques, which calls for more research utilizing network meta-analysis of diagnostic accuracy [97]. In other recent reviews, the differential diagnostic characteristics of seminomatous, non-seminomatous, and Leydig cell tumors are discussed in more detail [98,99].

## 5. Conclusions

To identify a neoplasm (from non-neoplasm) or malignant lesion (from benign lesion), existing studies utilized strain elastography (SE) by observing the color codes qualitatively or rating the lesions using a scoring system semi-quantitatively. In this review, there were fewer studies that evaluated the diagnostic accuracy of shear-wave elastography (SWE), though with fewer methodological variations. The pooled sensitivity for classifying malignant and benign lesions was 86.0% (95%CI, 79.7% to 90.6%). Nevertheless, as a result of substantial heterogeneity, the test performances of classifying neoplasm and non-neoplasm as well as the specificity of classifying malignant and benign were not concluded. Our subgroup analysis on the sonoelastography technique was unable to account for heterogeneity. All the included studies had a high risk of bias and applicability concerns. Future studies may consider utilizing multiparametric sonoelastography, deep learning, and ensemble learning to enhance the test performance.

## Figures and Tables

**Table 1:** Population information of the included studies.

Article	Sample Size	Mean Age	Sampling	Patient Source	Referral Indications
Aigner et al. [47] (2012)	50 PP/PT	42 (18–81)	Retrospective, consecutive	-	Patients with clinical suspicion of testicular tumor
Auer et al. [48] (2017)	55 PP/PT	39.5 (SD: 14.9)	Retrospective, consecutive	Radiology department	Evaluations of scrotal pain, scrotal abnormality, varicoceles, infertility, sexual development disorder, and F/U indetermined scrotal ultrasound findings.
Corcioni et al. [49] (2022)	* 78 PP/81 PT	34.3 (SD:12.2)^+^ 35.8 (SD:12.2)^−^	Prospective, consecutive	University hospital	Infertility, andrological screening, testicular pain
Goddi et al. [50] (2012)	88 PT	^#^ 34 (2 months–89 years)	Consecutive	Medical Center	Scrotal abnormalities
Konstantatou et al. [51] (2019)	86 PP/PT	Median: 36 (16–81)	Retrospective, consecutive	Hospital	Acute and nonacute scrotal conditions, indetermined focal intratesticular abnormality
Pozza et al. [52] (2016)	106 PP	34.5 (28–41.2)	Prospective, consecutive	Inpatients and outpatients referred to University	Infertility, andrological screening, F/U contralateral or ipsilateral tumor, testicular pain, varicocele, Klinefelter’s syndrome, F/U microlithiasis, elevated level of alpha-fetoprotein
Reginelli et al. [53] (2019)	54 PP/PT	Median: 42.2 (10–64)	Retrospective, consecutive	-	Clinical suspicion of testicular mass
Rocher et al. [54] (2019)	86 PP 89 PT	37.9 (SD: 13.2)	Prospective, consecutive	-	Infertility, pain, abnormal self-palpation, and others
Roy et al. [55] (2020)	! 338 PP 606 PT	43.2 (SD: 17.2, 17–78)	Prospective, consecutive	Ultrasound department	-
Schröder et al. [56] (2016)	67 PP 68 PT	Median: 39.8 (18–83)	Prospective, Consecutive	-	Suspicious testicular mass
Shaaban [57] (2017)	21 PP 23 PT	30 (18–54)	-	-	-

* Some patients had missing sonoelastography data; ! Reported sample size was contradictory to their data; ^#^ Information refers to data before exclusion; + true positive cases; − true negative cases; F/U: follow-up; PP: per-patient; PT: per-testis; SD: standard deviation.

**Table 2:** A summary of testicular or scrotal problems accounted by the included articles.

Non-Neoplastic (Non-Tumor)	Neoplastic (Tumor)	
	Benignity	Malignancy
Abscess Calcification Epidermoid or Dermoid/Cyst Epididymo-orchitis/Orchitis Focal inflammation Granuloma Hematoma Hydatid cyst Hydrocoele Infraction Ischemia Microlithiasis Pseudocyst Scarring/fibrosis Spermatoceles infection/hemorrhage	Adrenal rest tumor Leydig Cell tumor (benign) Sertoli Cell tumor (benign) Lipoma Papillary cystadenoma	Burned-out testicular tumor Embryonal carcinoma Germ cell tumor (includes seminoma and nonseminoma) Lymphoma Leydig Cell tumor (malignant) Sertoli Cell tumor (Malignant) Sex cord stromal tumor (Malignant) Metastasis from Adrenal tumor Mixed/nonseminoma Seminoma Teratoma

**Table 3:** Index test, measurement, and features of the included studies.

Article	Index Test (Instrument)			Measurements and Features
	Elastography	Other B-Mode US/Doppler	Comparison	
Aigner et al. [47]	SE: HI Vision EUB 8500	Sequoia 512	B vs. Doppler vs. SE	- B: lesion volume, echogenicity (hypo-, mixed echogenicity, hyper-). - Doppler: Vascularization (avascular, hypo-, hyper-). - SE: Tissue stiffness (soft, hard) based on color-codes and experience.
Auer et al. [48]	SE: HI Vision Ascendus	Logic E9	Doppler vs. CEUS vs. SE vs. (Doppler + SE) vs. (CEUS + SE)	- B: echogenicity (hypo-, mixed echogenicity). - Doppler: intralesional Doppler signal. - CEUS: lesion vascularity. - SE: Tissue stiffness (soft, hard) based on color-codes.
Corcioni et al. [49]	SE: Aplio 500	Esaote MyLab 70 Gold XVG	SE vs. CEUS	- B: location, diameter, echogenicity (hypo-, hyper-, heterogeneous), lesion margin (sharp, irregular). - Doppler: hypervascularization. - CEUS: vascularization. - SE: tissue stiffness (soft, intermediate, hard) based on color-codes.
Goddi et al. [50]	SE: HI Vision EUB 8500	Preirus	B + SE + Doppler	- B: lesion shape (nodular, pseudo-nodular), size (<5 mm, 6–10 mm, >11 mm). - Doppler: peripheral vascularization. - SE: Itoh’s 5-point elastographic score (cut-off > 3).
Konstantatou et al. [51]	SE: Hitachi HV900	Siemens S2000	B vs. Doppler vs. SE	- B: echogenicity (cystic, hypo-, iso-/hype-, heterogenous), microlithiasis. - Doppler: vascularization (avascular, hypo-, iso-/hyper-). - SE: strain ratio, Yusuf’s 6-point visual elastographic score (cut-off > 3).
Pozza et al. [52]	SE: Philips IU22	-	B vs. SE	- B: lesion size, hypoechogenicity, parenchymal microlithiasis, intralesional vascularization. - SE: strain ratio (cut-off determined by ROC analysis), Itoh’s 5-point elastographic score (cut-off > 3).
Reginelli et al. [53]	SE: Hi-Vision Preirus	-	SE vs. (B + Doppler) vs. (B + Doppler + SE)	- B: lesion size, homogeneity, echogenicity (hypo-, iso-, hyper-), pseudo-capsule appearance. - Doppler: vascular signal (hypo-, iso- or hyper-). - SE: tissue stiffness (soft, medium, hard) according to chromatic value.
Rocher et al. [54]	SWE: Aixplorer	Aplio 500	SWE vs. (B + Doppler) vs. (B + Doppler + SWE)	- B: number of nodule (null, unique, >1, diffuse), echogenicity (hyper-, iso-, hypo-, heterogeneous), calcification, boundaries. - Doppler: vascularization. - SWE: mean stiffness & SD of lesion (*E*_mean_), stiffness of the stiffness area (*E*_max_), stiffness of normal parenchyma (*E*_ref_), *E*_mean_/*E*_ref_, *E*_max_/*E*_ref_.
Roy et al. [55]	SWE: Aplio 500	-	SWE	- SWE: degree of filling of ROI in a 3-point scale, stiffness (cut-off determined by ROC analysis).
Schröder et al. [56]	SE: -	Philips iU-22	SE vs. (B + Doppler)	- B: lesion size. - SE: Q-size > 1.05, Modified Itoh’s 5-point elastographic score, elasticity score difference between lesion and healthy tissue, strain ratio (cut-off determined by ROC analysis).
Shaaban [57]	SE: Hitachi Hi Vision Avius	-	B + Doppler + SE	- B: lesion volume, echogenicity. - Doppler: vascularization. - SE: Tissue stiffness based on color-coded pattern and experience

B: B-mode; SE: strain elastography; SWE: shear-wave elastography; US: ultrasound.

**Table 4:** Reference test and operator information for index tests (blinding, number of radiologists, and their experience) of the included studies.

Article	Reference Test		No. of Radiologist (yr. exp)	Blind
	Test for (+)	Test for (−)		
Aigner et al. [47]	HPA	Benign: F/U in 6 weeks & 3 months. Inflammation: F/U after 2–3 days & then weekly Infarction: F/U within 24 h.	1 (>5)	YES
Auer et al. [48]	HPA	Sonographic F/U within the 1st 2–3 days, then weekly up to 6 week, & after 3 months	2 (>10 in SE)	YES
Corcioni et al. [49]	HPA, patients declined HPA underwent F/U at least 2 years.		1 (>10)	-
Goddi et al. [50]	HPA	F/U every 3 or 6 months	3 (>20 in US)	-
Konstantatou et al. [51]	HPA	F/U until resolution or non-progression was documented	2 (6 & 7 in SE)	YES
Pozza et al. [52]	Biomarkers (human chorionic gonadotropin, placental alkaline phosphatase, alpha-fetoprotein, carcinoembryonic antigen, ferritin, lactate dehydrogenase)		2 (>5)	YES
	HPA, patients declined HPA underwent F/U every 3 months for a min of 18 months	Repeated F/U (6 consecutive scans)		
Reginelli et al. [53]	Biomarkers (alpha-fetoprotein, beta-human chorionic gonadotropin)		1 (15)	-
	Nodules > = 2 cm: HPA. Nodules < 2 cm with malignant pattern: Inguinoscrotal exploration	F/U over time		
Rocher et al. [54]	HPA	Clinical tests (17-hydroxyprogesterone, C-reactive protein, leukocytes)	1 (>20 in US)	-
Roy et al. [55]	History, clinical tests, B-mode and Doppler ultrasound, recovery after antibiotic treatment, abscesses confirmed surgically, no modification of ultrasound on F/U > 1 year		2 (6 & 20)	-
Schröder et al. [56]	HPA		1 (-)	-
Shaaban [57]	HPA	F/U up to one month	1 (8 in SE)	-

HPA: surgical exploration with histopathological analysis; F/U: follow-up; SE: strain elastography; US: ultrasound; yr. exp: years of experience.

**Table 5:** Test performance of the included studies.

Article	Classification	Modality	Evaluation Metrics and Outcomes						
			Acc	Sn/Rc	Sp	PPV/Pc	NPV	AUC	Others
Aigner et al. [47]	Tumor vs. non-tumor	SE	94%	100%	81%	92%	100%	-	-
		B	92%	100%	75%	89%	100%	-	-
Auer et al. [48]	Malignant vs. benign	SE	78.2%	100%	72.1%	-	-	-	-
		Doppler	83.6%	66.7%	88.4%	-	-	-	-
		CEUS	81.8%	100%	76.7%	-	-	-	-
		SE + Doppler	89.1%	66.7%	95.3%	-	-	-	-
		SE + CEUS	94.5%	100%	93.0%	-	-	-	-
Corcioni et al. [49]	Malignant vs. benign	SE	-	66.7%	-	-	-	-	-
	LCT vs. non-LCT		-	36.0%	-	-	-	-	-
	LCT vs. non-LCT	CEUS	-	96.9%	94.0%	-	-	0.954	DOR: 480.5
Goddi et al. [50]	Malignant vs. benign	SE: VES	95.8%	87.5%	98.2%	93.3%	96.4%	-	-
Konstantatou et al. [51]	Malignant vs. benign	Doppler	-	77.4%	81.8%	-	-	-	-
		SE: SR	-	74.2%	70.9%	-	-	0.722	-
		SE: VES	58.1%	80.7%	45.5%	45.5%	80.7%	0.620	-
	Neoplastic vs. non-neoplastic	Doppler	-	68.8%	97.4%	-	-	-	-
		SE: SR	-	68.8%	81.6%	-	-	0.730	-
		SE: VES	70.9%	81.3%	57.9%	70.9%	71.0%	0.715	-
Pozza et al. [52]	Malignant vs. benign	B	-	89.2%	85.5%	76.7%	93.7%	0.878	-
		SE: SR	-	59.4%	66.6%	48.9%	75.4%	0.631	-
		SE: VES	-	81.1%	79.7%	68.2%	88.7%	0.804	-
	Neoplastic vs. non-neoplastic	B	-	94.6%	87.1%	94.7%	87.1%	0.910	-
		SE: SR	-	69.3%	61.3%	81.2%	45.2%	0.653	-
		SE: VES	-	58.7%	100%	100%	50%	0.793	-
Reginelli et al. [53]	! Malignant vs. benign	B + Doppler	81.0%	86.0%	64.0%	84.0%	48.0%	-	-
		SE	87.0%	85.0%	78.0%	93.0%	71.0%	-	-
		B + Doppler + SE	90.0%	100%	83.0%	91.0%	100%	-	-
Rocher et al. [54]	(Malignant + Burnout tumor) vs. benign LCT	B + Doppler: size	77.9%	67.3%	96.4%	97.1%	62.8%	0.88	-
		SWE: SD	79.8%	93.9%	62.5%	75.4%	89.3%	0.77	-
		B + Doppler + SWE: #	86.5%	95.9%	75.0%	82.5%	93.8%	0.93	-
	* (Malignant + Burnout tumor) vs. (Benign LCT + other benign tumors)	B + Doppler: Calcification score	74.2%	55.1%	97.5%	96.4%	63.9%	0.85	-
		SWE: SD	79.8%	93.9%	62.5%	75.4%	89.3%	0.77	-
		B + Doppler + SWE: #	86.5%	95.9%	75.0%	82.5%	93.8%	0.91	-
Roy et al. [55]	! Tumor vs. non-tumor	SWE: ROI filling	-	82%	81%	85%	98%	0.881	-
Schröder et al. [56]	Neoplastic vs. non-neoplastic	B	88.2%	100%	42.9%	87.1%	100%	-	-
		Doppler	82.1%	81.1%	85.7%	95.6%	54.5%	-	-
		SE: VES	84.6%	98.1%	25.0%	85.2%	75.0%	-	-
		SE: ΔVES	89.1%	97.8%	50.0%	89.8%	83.3%	-	-
		SE: SR	81.1%	90.5%	45.5%	86.4%	55.6%	-	-
		B + SE: Q-size	61.1%	56.7%	83.3%	94.4%	27.8%	-	-
		CEUS	91.0%	92.6%	84.6%	96.2%	73.3%	-	-
Shaaban [57]	! Neoplastic vs. non-neoplastic	B + Doppler + SE	-	100%	40%	37.5%	100%	-	-

Acc: Accuracy: AUC: area under ROC curve; B: B-mode; CEUS: contrast-enhanced ultrasound; DOR: diagnostic odds ratio: LCT: Leydig cell tumor; NPV: negative predictive value; Pc: precision; PPV: positive predictive value; Rc: Recall; SD: standard deviation; Sn: sensitivity; Sp: specificity; SR: strain ratio; SWE: shear-wave elastography; SE: strain elastography (those without specifying parameters were determined based on the color codes qualitatively); VES: visual elastography score. * This classification scheme of benignity and malignancy was chosen for the meta-analysis in this study. ! Reported outcomes were contradictory to their data. # SWE Parameters for classification included peripheral vascularization, ratio of highest stiffness value to the stiffness of adjacent normal parenchyma and grouped microliths.

**Table 6:** The overall summary and subgroup estimates for the meta-analyses.

Comparison	Subgroup Analysis	Pooled Sensitivity (95% CI)	Pooled Specificity (95% CI)	Pooled DOR (95% CI)
Non-neoplasm vs. Neoplasm	Summary	92.1% (62.8% to 98.7%)	79.2% (26.2% to 97.6%)	44.21 (5.48 to 356.79)
	SE color code	-	-	-
	SE scoring system	84.4% (58.5% to 95.4%)	72.1% (13.9% to 97.6%)	14.01 (2.10 to 93.46)
	SWE	-	-	-
Benignity vs. Malignancy	Summary	86.0% (79.7% to 90.6%)	82.4% (60.4% to 93.5%)	28.71 (9.60 to 85.88)
	SE color code	86.% (65.9% to 95.3%)	82.2% (57.0% to 94.1%)	26.77 (7.14 to 115.84)
	SE scoring system	83.2% (73.5% to 89.8%)	84.6% (43.3% to 97.5%)	27.22 (2.81 to 263.27)
	SWE	-	-	-

CI: confidence interval; DOR: diagnostic odds ratio; SE strain elastography; SWE: shear-wave elastography.

## Data Availability

Not applicable.
